# FK228-Mediated Restoration of Tight Junction Proteins in Diabetic Neuropathy

**DOI:** 10.3390/biology15151257

**Published:** 2026-07-31

**Authors:** Eileen Chen, Vikram Thakur, Erina Chowdhury, Amogh Misra, Carlos Valdez, Munmun Chattopadhyay

**Affiliations:** 1 Paul L Foster School of Medicine, Texas Tech University Health Sciences Center El Paso, 130 Rick Francis Street, El Paso, TX 79905, USA; chen.y.eileen@gmail.com (E.C.); amogh.misra@ttep.edu (A.M.); 2 Department of Molecular and Translational Medicine, Texas Tech University Health Sciences Center El Paso, 130 Rick Francis Street, El Paso, TX 79905, USA; vikram.thakur@ttep.edu (V.T.); carlos.valdez@ttep.edu (C.V.); 3 Francis Graduate School of Biomedical Sciences, Texas Tech University Health Sciences Center El Paso, 130 Rick Francis Street, El Paso, TX 79905, USA; erinachowdhury422@gmail.com

**Keywords:** diabetes, neuropathy, tight junction, dorsal root ganglia, FK228

## Abstract

Diabetic painful neuropathy (DPN) is a common complication of diabetes. Despite ongoing efforts, the underlying biological mechanisms of DPN remain poorly understood. Hyperglycemia-mediated oxidative stress can affect cell barrier properties, triggering low-grade inflammation. Previous studies have reported increased histone deacetylase (HDAC) activity and altered expression of tight junction proteins in diabetes, which may link to nerve damage. This study examined the effects of the HDAC inhibitor FK228 on cell barrier-mediated changes in the spinal cord and dorsal root ganglia (DRG) of type 2 diabetic mice. Diabetic mice were treated with FK228 (1 mg/kg, twice a week for 3 weeks). Behavioral assessment of cold hypersensitivity was assessed at the end of the treatment regimen. In vitro studies were conducted using ND7/23 immortalized DRG cells. The expression of tight junction proteins (occludin, claudin-1, and zona occludens-1 or ZO-1) and a number of stress-related cellular markers, including HDAC2, growth-associated protein (GAP) 43, epidermal growth factor receptor (EGFR), and nuclear factor erythroid 2-related factor (Nrf2), were evaluated in DRG, spinal cord tissue, and cultured ND7/23 DRG cells following treatment. FK228 treatment demonstrated changes in cold pain sensitivity as well as the expression of tight junction proteins and stress related markers in both in vivo and in vitro models of diabetic neuropathy. These findings indicate that HDAC inhibition by FK228 may support the maintenance of tight junction protein expression and alter cellular stress responses in the spinal cord and DRG, suggesting a possible role of FK228 in mitigating DPN.

## Simple Summary

Diabetic painful neuropathy (DPN) is a common and distressing complication of diabetes and chronic high blood glucose levels may induce damage to the peripheral nerves, but the underlying mechanisms are not fully understood. Elevated blood sugar can increase oxidative stress and inflammation, which may weaken the protective barriers around nerves. This study investigated whether FK228, a drug that blocks histone deacetylases (HDACs), could help reduce these harmful changes. To understand the process, type 2 diabetic mice were treated with FK228, and cultured sensory neuronal cells were also tested with the drug under high glucose condition. This investigation examined pain sensitivity and measured proteins involved in maintaining nerve barriers and protecting cells from stress. The findings indicated that FK228 changed pain-related behaviors and altered the levels of proteins involved in tight junctions and cellular stress in the spinal cord, dorsal root ganglia, and cultured neuronal cells. These observations suggest that blocking HDAC activity with FK228 may help protect nerve tissues and improve the expression of the cell barrier proteins as well as reducing cellular stress, thereby making it a potential therapeutic approach for diabetic painful neuropathy.

## 1. Introduction

Diabetic painful neuropathy (DPN) is among the most prevalent complications of long-term diabetes, affecting more than 50% of patients and significantly impairing quality of life [1,2]. Despite its high prevalence, current therapeutic options remain largely symptomatic, with no effective treatments available to prevent or reverse neuropathy or provide complete pain relief [3,4]. Emerging neuromodulatory approaches, including spinal cord and peripheral nerve stimulation, have shown promise; however, substantial gaps remain in targeting the underlying mechanisms responsible for disease progression [5]. Consequently, there is a critical need to identify novel therapeutic strategies that address the molecular and cellular pathways involved in diabetic neuropathy. Accumulating evidence suggests that epigenetic regulation, particularly through histone deacetylases (HDACs), plays an important role in axonal regeneration, oxidative stress, inflammation, and neuropathic pain [6,7,8]. Previous studies from our laboratory demonstrated that treatment with the HDAC inhibitor FK228 (Romidepsin) alleviates neuropathic pain in a type 2 diabetic (T2D) animal model [9]. The current investigation also shows that diabetic animals exhibited pain related behavior as assessed by cold allodynia, which was alleviated by the FK228 treatment. However, the mechanisms underlying these protective effects are still not completely understood.

One potential contributor to DPN pathogenesis may involve with the disruption of specific neuronal barriers. High blood glucose levels as well as tissue injury have been shown to alter the expression and organization of tight junction proteins, including occludin, claudins, and the scaffolding proteins zona occludens (ZO)-1, ZO-2, and ZO-3 [10,11]. Hyperglycemic condition may exacerbate neural function and facilitate infiltration by pro-inflammatory mediators, as previously shown by our laboratory [9]. In addition, impaired neuronal regeneration is a characteristic of diabetic neuropathy. Growth-associated protein 43 (GAP43), a key regulator of neuronal growth and axonal regeneration, is reduced in experimental models of diabetes and has been linked to diminished regenerative capacity [12,13]. To further assess the neuron-specific effects of FK228, it is important to understand neuronal stability using the neuron-specific markers. Oxidative stress is another major driver of diabetic neuropathy. Nuclear factor erythroid 2-related factor 2 (Nrf2), a master regulator of cellular antioxidant defenses, has been implicated in neuroprotection and treatment with FK228 may contribute to the beneficial effects on this transcriptional regulator in diabetic neuropathy [14,15]. Furthermore, epidermal growth factor receptor (EGFR) signaling, which regulates cellular growth, proliferation, and survival, has emerged as a potential mediator of neuropathic pain, as EGFR inhibition has been associated with reduced pain-related behaviors in animal models [16,17,18]. Given the multifactorial nature of DPN, a better understanding of the mechanisms prompting the changes in cell junctions and neuronal atrophy is needed. Therefore, the present study investigated the effects of FK228 on tight junction protein expression, neuronal markers, oxidative stress pathways, and EGFR signaling in the spinal cord and dorsal root ganglia (DRG) of diabetic animals. These observations may contribute to a better understanding of the changes in cell junction protein expression associated with HDAC inhibition in diabetic neuropathy and may provide potential therapeutic targets for DPN.

## 2. Materials and Methods

### 2.1. In Vitro Study

ND7-23 (rat DRG/mouse neuroblastoma hybrid) cell line expressing the rat Nav1.8 was used (Millipore/Sigma, St. Louis, MO, USA) for in vitro studies. The cell line was screened by the manufacturer using the MycoSensorTM PCR Assay Kit (Agilent Technologies, Inc., Santa Clara, CA, USA) to confirm the absence of Mycoplasma species. The cells were cultured in neurobasal medium with necessary supplements. Once confluent, the cells were divided into three groups: Normal Glucose (NG) and High Glucose (HG) and HG with FK228 treatment. For the HG group, 25 mM glucose was added for 24–48 h. After twenty-four hours of glucose exposure, a group of HG cells was treated with 50 nM of FK228 overnight. The dose of FK228 was calculated based on earlier studies and a dose response study in the lab. The following day, samples from all three groups of cells were harvested for further analysis. The experiments were repeated twice, and each group of cells was used from three independent cell culture wells as biological replicates.

### 2.2. Animal Study and Ethical Approval

The study was performed in accordance with ARRIVE guidelines. The study utilized ob/ob mice with homozygous leptin mutation (B6.Cg-Lepob/J), an established model closely resembling type 2 diabetes (T2D) in humans. Naïve control mice with a C57BL/6J background served as the control group. All animals were obtained from The Jackson Laboratory, Bar Harbor, ME, USA. The animals were housed in groups in the SPF facility of Laboratory Animal Resource Center (LARC) at TTUHSC, El Paso with the recommended light/dark cycle, humidity and temperature. The lean control and ob/ob mice were provided ad libitum diet and water during the entire experimental timeline. All procedures were conducted in accordance with approved IACUC protocol (Protocol # 14035). The lean control and ob/ob mice were acclimatized for a week once received and blood glucose was measured weekly. Animals with a blood glucose level of 250 mg/dL or more were included in the study as diabetic at approximately 8 weeks of age, whereas animals with a blood glucose level of less than 200 mg/dL were excluded and considered not diabetic.

### 2.3. Randomization and Treatment

Four weeks after the onset of diabetes, the diabetic mice were randomly assigned in 2 groups; one group was injected with HDAC-inhibitor, FK228 (Romidepsin, Selleck Chemicals; Houston, TX, USA) intraperitoneally for twice a week for 3 weeks at a dose of 1 mg/kg. The dose was calculated based on the previous animal study [19]. A total of 24 animals were randomized into three groups: lean control, diabetic alone, and diabetic with FK228 treatment. For the diabetic and treated group, 4–6 animals were used per group as some were excluded due to diabetes-related health issues. The diabetic and treated animals were frequently monitored throughout the study timeline, and the cages were changed as needed. To minimize potential bias, investigators were blinded to the treatment regimen during both data collection and subsequent analysis. Behavioral studies were performed to measure pain related changes at the end of the 3-week treatment regimen. The animals from all groups were then euthanized in accordance with the approved IACUC policy and AVMA 2020 guidelines for CO_2_ euthanasia with a displacement rate of 30 to 70% of the chamber volume with carbon dioxide per minute followed by bilateral thoracotomy to assure euthanasia. The tissue and blood samples were collected once the euthanasia was confirmed for further analysis of cellular markers of cell junction proteins and neuronal and stress mediators. The study was performed in accordance with ARRIVE guidelines.

### 2.4. Behavioral Study: Cold Allodynia

All mice were acclimatized for 20–30 min every day for 5 days prior to testing. The mice were placed on a mesh floor 18 inches above the table. On the day of testing, the animals were kept on the mesh until they were stable and calm, then 0.1 cc of acetone was sprayed onto the plantar surface of their hind paws using a 1 cc syringe. The paw withdrawal latency was assessed either as flinching or licking with a cut off limit at 30 s. A total of 3 responses from each animal were assessed at 5 min intervals by a blinded observer.

### 2.5. Statistical Analysis

For statistical evaluation, group comparisons were conducted using one-way analysis of variance (ANOVA), followed by post hoc analysis utilizing Bonferroni’s multiple comparison tests. The data were further analyzed using SPSS software (Systat version 13.0, SPSS Inc., Chicago, IL, USA) with a significance threshold level set at *p* < 0.05 was used. Results are presented as mean ± standard error of the mean (SEM).

### 2.6. Western Blot Analysis

The DRG and spinal cord were freshly harvested from each animal, and the tissues were homogenized with lysis buffer. For cell culture studies, cells were scraped and collected in lysis buffer. Protein was isolated and quantified for each sample, and once the protein estimation was complete, Western blot analysis was performed. Western blot analysis was also conducted on the samples derived from the control, diabetic, and diabetic+FK228 animal groups (*n* = 3–6 mice, and due to small size of DRGs, they are often pooled from 2 animals) and on 3 groups of the cultured neuron-derived immortal ND7/23 cell (*n* = 3 wells/group), as described in our earlier studies [9]. Primary antibodies targeting HDAC2 (Catalog # 5113), NeuN (Cat. # 24307; 1:1000 dilution; Cell Signaling, Danvers, MA, USA), ZO-1 (Cat. # 21773-1-AP; 1:500 dilution; Proteintech, Rosemont, IL, USA) claudin-1 (1:500 dilution, Cat. # PA5-20754), occludin (1:500 dilution, Cat. # 33-1500), pNrf2 (Cat. # PA5-67520, 1:400 dilution; ThermoFisher, Waltham, MA, USA), EGFR (Cat. # SC-03, 1:400 dilution; Santa Cruz Biotechnology Inc., Dallas, TX, USA) and the secondary antibody anti-rabbit IgG or anti-mouse IgG (1:3000 Amersham, Piscataway, NJ, USA) were used and imaged using Pierce ECL Western Blotting Substrates (1:1 ratio; ThermoFisher, Waltham, MA, USA). Housekeeping genes β-actin (Cat. # A5441; 1:2000; Millipore Sigma, St. Louis, MO, USA) or GAPDH (Cat. # 2118; 1:2000; Cell Signaling Technology, Inc. Danvers, MA, USA) were used as internal loading controls. We used either of these housekeeping genes to avoid the issues of antibody specific re-probing of the blots. The intensity of chemiluminescence signal for each band was assessed using a PC-based image analysis system (ChemiDoc XRS System, Bio-Rad Laboratories, Hercules, CA, USA). Data were expressed as mean ± SEM; *n* = 3–6 animals per group.

### 2.7. Immunohistochemistry

The harvested tissues were fixed with fresh 4% PFA for 72 h and then placed in 30% sucrose in phosphate-buffered saline (PBS) for cryoprotection. The tissues were next embedded in O.C.T. Compound (ThermoFisher, Waltham, MA, USA) and later cryo-sectioned on super-frost slides. The sections were again fixed with fresh 4% PFA for 30 min, washed and blocked with PBS with 1% normal goat serum and 0.3% Triton X-100 (Bio-Rad Laboratories, Hercules, CA, USA) for one hour. This was followed by one wash with PBS before incubation with specific primary antibodies targeting claudin-1 (1:800 dilution), MAP2 (Cat. # MA5-12826; 1:2000 dilution; ThermoFisher, Waltham, MA, USA), ZO-1 (1:800 dilution; Proteintech, Rosemont, IL, USA), HDAC2, MAP2 (1:2000 dilution; Catalog # 4542), GAP43 (Cat. # 8945, 1:400 dilution; Cell Signaling Technology, Inc. Danvers, MA, USA), and Tuj-1 (Cat. # MAB1195, 1:2000 dilution; R&D Systems, Inc. Minneapolis, MN, USA), as previously reported [20]. The Alexa fluor 594 and Alexa fluor 488 conjugated secondary antibodies (ThermoFisher, Waltham, MA, USA) were diluted 1:2000. After the secondary antibody incubation, the slides were washed 3 times with PBS and stained with DAPI for 3–4 min. After the final wash with PBS, Fluoromount G (Electron Microscopy Sciences, Fort Washington, PA, USA) mounting media was used to mount the slides. The cells cultured on the coverslips were also processed in the same manner. The images were captured using a Nikon Eclipse Ni-E microscope (Nikon Instruments Inc., Melville, NY, USA).

### 2.8. Morphometric Analysis

The images of the immunostained tissues or cells were captured using a Nikon Eclipse Ni-E microscope and the analyzed using the NiS Elements computer-based image analysis system (Nikon Instruments Inc. Melville, NY, USA). A researcher blinded to the treatment group performed the analysis and three different areas of the tissue sections from for each animal were investigated.

## 3. Results

### 3.1. Effect of FK228 on HDAC2 Expression and GAP43 Levels in the ND7/23 DRG Cells

**Figure 1:**
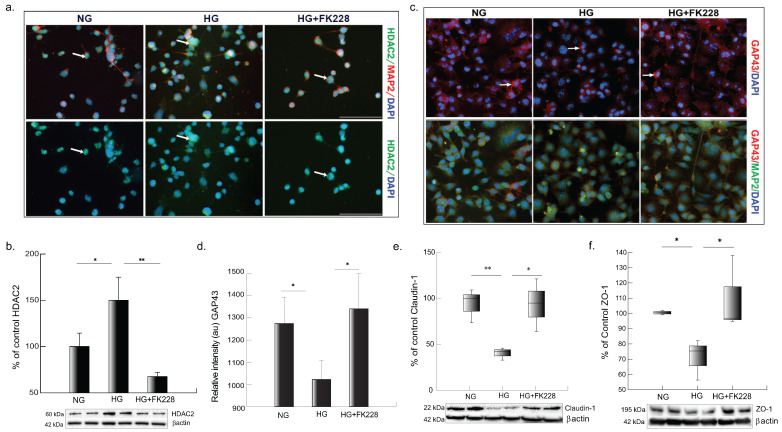
(**a**) Immunocytochemistry of HDAC2 and neuron-specific protein marker MAP2 revealed higher HDAC2 immunoreactivity in hyperglycemic DRG cells that was significantly reduced in hyperglycemic ND7/23 sensory neuronal cells treated with FK228. Bar = 100 µm. White arrows denote the expression of HDAC2. (**b**) Hyperglycemic ND7/23 DRG cells (HG) demonstrated increase in HDAC2 expression compared to normoglycemic cells (NG) and overnight treatment of FK228 showed significant reduction in HDAC2 expression in HG+FK228 cells with a total of 42 h hyperglycemia as assessed by Western blot analysis. (*n* = 3/group; * *p* < 0.05, ** *p* < 0.01). (**c**,**d**) To examine the possible axonal growth and plasticity, GAP43 immunocytochemistry showed a significant decrease in immunoreactivity under hyperglycemic condition compared to normoglycemic ND7/23 neuronal DRG cells (* *p* < 0.05). HDAC inhibitor treatment exhibited a substantial increase in GAP43 expression (* *p* < 0.05). White arrows denote the expression of GAP43. (**e**). Hyperglycemic DRG cells in culture also demonstrated a considerable decrease in Claudin-1 expression, and restoration of Claudin-1 expression was observed after overnight treatment with 50 nM FK228 (** *p* < 0.01; * *p* < 0.05). (**f**) Treatment with 50 nM FK228 to hyperglycemic ND7/23 DRG cells displayed a significant increase in ZO-1 expression in the DRG cells as shown by Western blot analysis (* *p* < 0.05).

Consistent with our previous animal studies, HDAC2 expression in ND7/23 DRG cells under hyperglycemic conditions was increased compared to normoglycemic controls. To further assess HDAC2 expression in cultured DRG cells, immunocytochemistry was performed using antibodies against HDAC2 and the neuron-specific marker microtubule-associated protein-2 (MAP2). Under hyperglycemic conditions, increased HDAC2 immunoreactivity was observed across neuronal cells. Treatment with FK228 was associated with significant reduction in HDAC2 expression in hyperglycemic cells, as confirmed by both immunocytochemical and Western blot analysis (** *p* < 0.01, * *p* < 0.05; Figure 1a,b and Supplementary Figure S1b). In addition, GAP43 expression was evaluated as a marker associated with neuronal growth and plasticity. Quantification of relative intensity revealed that GAP43 levels were significantly decreased under hyperglycemic conditions compared to normoglycemic ND7/23 DRG cells (* *p* < 0.05). Following FK228 treatment, an increase in GAP43 expression was observed (* *p* < 0.05; Figure 1c,d), suggesting a potential effect on pathways related to axonal growth and plasticity under hyperglycemic conditions.

### 3.2. FK228 Treatment Improved Cold Allodynia and Restored NeuN Expression in the DRG of Diabetic Mice

**Figure 2:**
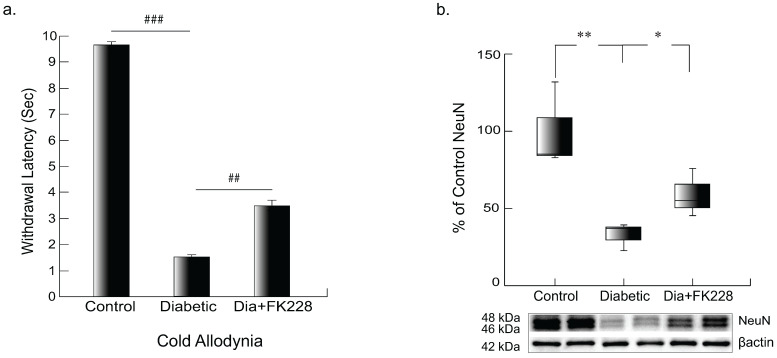
(**a**) The diabetic ob/ob mice developed cold allodynia compared to their control counterparts whereas it was alleviated after 3 weeks of FK228 treatment as assessed by withdrawal latency for acetone spray test (*n* = 4–6; ### *p* < 0.0001, ## *p* < 0.001). (**b**) Downregulation of NeuN expression in the DRG of the diabetic mice was significantly attenuated after FK228 treatment (** *p* < 0.01, * *p* < 0.05; *n* = 3).

Diabetic painful neuropathy was assessed in the ob/ob diabetic mice by withdrawal latency for acetone spray test for cold allodynia. The diabetic mice treated with FK228 showed a marked alleviation of cold allodynia, while untreated diabetic mice exhibited significant cold allodynia (### *p* < 0.0001, ## *p* < 0.001; Figure 2a). A substantial reduction in neuronal marker NeuN expression was observed in diabetic DRG, which was significantly attenuated after FK228 treatment (** *p* < 0.01, * *p* < 0.05; Figure 2b and Supplementary Figure S2b).

### 3.3. FK228 Treatment Improved Claudin-1 and Occludin Expression in the Spinal Cord of Diabetic Mice and in the Hyperglycemic DRG Cells

**Figure 3:**
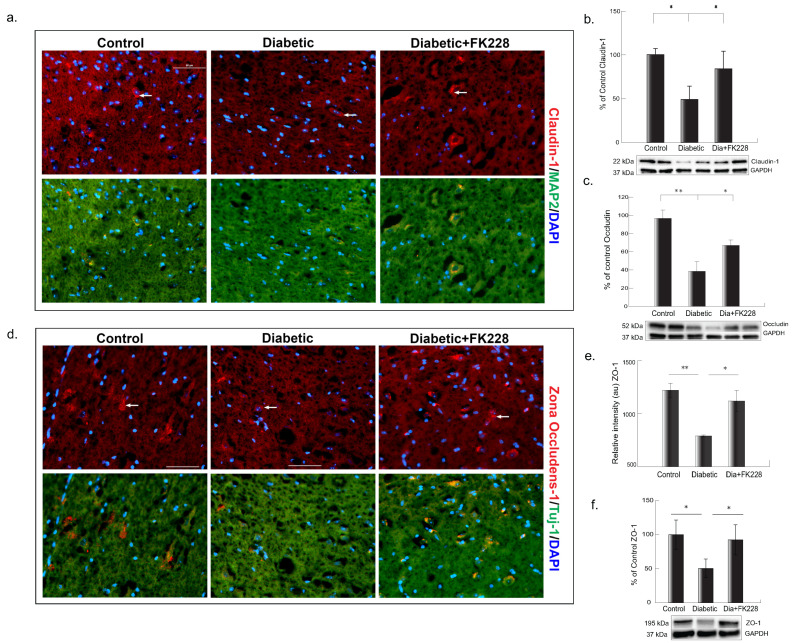
(**a**,**b**) Claudin-1 expression in the spinal cord showed a significant decrease in diabetic animals with 7–8 weeks post-diabetes when compared to their age-matched controls (* *p* < 0.05); 3 weeks treatment with the FK228 altered Claudin-1 levels, as confirmed by Western blot and immunohistochemistry. Bar = 50 µm. White arrows denote the expression of Claudin-1. (**c**) Occludin expression in the spinal cord was significantly reduced in 7–8 weeks post-diabetic animals compared to age-matched non-diabetic controls (* *p* < 0.05, ** *p* < 0.01), and 3 weeks treatment with FK228 treatment improved occludin expression levels. The data presented in the graph indicates mean ± SEM, *n* = 3–4 mice/group. (**d**,**e**) Immunohistochemical study of ZO-1 expression in spinal cord tissue also demonstrated a robust decrease in ZO-1 expression in 7–8-week post-diabetic animals; FK228 treatment for 3 weeks exhibited clear improvement in ZO-1 expression in diabetic spinal cord. Bar = 50 µm (*n* = 3 mice/group; ** *p* < 0.01, * *p* < 0.05). White arrows denote the expression of ZO-1. (**f**) Western blot analysis of DRG from 7–8-week post-diabetic mice revealed a significant decrease in ZO-1 expression; treatment with FK228 for 3 weeks showed an increase in ZO-1 expression in the DRG of diabetic mice (* *p* < 0.05; *n* = 3 mice/group).

To further examine the effects of FK228 on tight junctions in spinal cord neurons, we evaluated the expression of claudin-1. Claudin-1 levels were considerably decreased in diabetic animals compared to non-diabetic controls (* *p* < 0.05). Following FK228 treatment, claudin-1 expression was increased, as observed by Western blot analysis (* *p* < 0.05; Figure 3b and Supplementary Figure S3b). The immunohistochemical studies also supported these changes after treatment, where the marker MAP2 elucidated neuronal colocalization (Figure 3a). A similar pattern was observed in ND7/23 DRG cells, where hyperglycemia significantly decreased claudin-1 expression and FK228 treatment was associated with substantial increase in claudin-1 expression in these hyperglycemic ND7/23 DRG cells (** *p* < 0.01, * *p* < 0.05; Figure 1e and Supplementary Figure S1e). Another tight junction protein, occludin expression, was evaluated in the spinal cord of diabetic animals. A robust reduction of occludin expression in the diabetic mice was observed compared to control counterparts (** *p* < 0.01). Treatment with FK228 was associated with a considerable change in occludin levels in diabetic animals, as shown by Western blot analysis (* *p* < 0.05; Figure 3c and Supplementary Figure S3c), suggesting a potential effect on tight junction-related processes under hyperglycemic conditions.

### 3.4. Improved ZO-1 Expression in the Hyperglycemic DRG Cells and Spinal Cord of Diabetic Mice After FK228 Treatment

To extend our understanding of the hyperglycemia-mediated changes in tight junction-associated scaffold protein ZO-1, hyperglycemic ND7/23 DRG cells were analyzed, which demonstrated a significant decrease in ZO-1 expression, whereas FK228 treatment corresponded with a stable increase in ZO-1 expression (* *p* < 0.05; Figure 1f and Supplementary Figure S1f). Immunohistochemical analysis of spinal cord tissue from diabetic mice also showed significant reduction in ZO-1 expression as confirmed by relative intensity study, while treatment with FK228 was associated with considerably higher expression of ZO-1 levels whereas the neuronal colocalization was verified with Tuj-1 staining (** *p* < 0.01, * *p* < 0.05; Figure 3d,e), suggesting a potential effect on tight junction-related changes under diabetic conditions. To further assess changes in tight junctions under diabetic conditions, ZO-1 expression was evaluated in DRG neurons of diabetic animals, where a robust decrease in ZO-1 expression was observed (* *p* < 0.05). FK228 treatment was associated with a substantial increase in ZO-1 levels, as indicated by Western blot analysis (* *p* < 0.05; Figure 3f and Supplementary Figure S3f).

### 3.5. FK228 Treatment Was Associated with Changes in EGFR Expression and Activation of Nrf2 in Diabetic DRG and Spinal Cord

**Figure 4:**
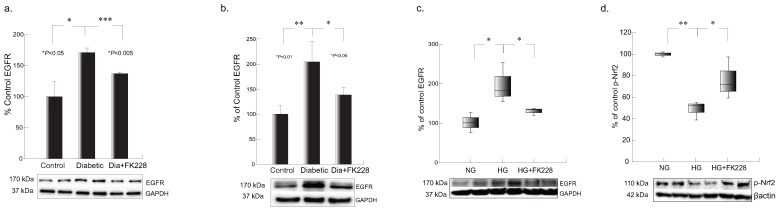
(**a**) Downregulation of EGFR expression in the spinal cord of the diabetic mice was significantly attenuated after FK228 treatment (*** *p* < 0.005, * *p* < 0.05; *n* = 3). (**b**,**c**) EGFR expression was significantly increased in the DRG of the diabetic mice as well as in the hyperglycemic ND7/23 DRG cells, and treatment with FK228 inhibited the increase in EGFR expression in both *in vivo* and *in vitro* conditions as confirmed by Western blot analysis (** *p* < 0.01, * *p* < 0.05, *n* = 3/group). (**d**) The transcription factor Nrf2 activation was substantially downregulated in DRG cells under hyperglycemic condition whereas HDAC inhibition had significant beneficial effects on Nrf2 activation under hyperglycemia (** *p* < 0.01, * *p* < 0.05; *n* = 3).

A substantial increase in EGFR expression was observed in diabetic spinal cord, which was significantly attenuated after FK228 treatment (*** *p* < 0.01, * *p* < 0.05; Figure 4a and Supplementary Figure S4a). EGFR expression was also significantly increased in DRG sensory neurons of diabetic mice compared to control mice, while FK228 treatment was associated with a substantial reduction in EGFR expression (** *p* < 0.01, * *p* < 0.05; Figure 4b and Supplementary Figure S4b). A similar pattern was observed in hyperglycemic ND7/23 DRG cells, where EGFR expression was notably increased (* *p* < 0.05; Figure 4c and Supplementary Figure S4c). The transcription factor nuclear factor erythroid 2-related factor 2 (Nrf2), which is involved in cellular responses to oxidative stress, was also evaluated. Phosphorylated Nrf2 (pNrf2) levels were remarkably decreased under hyperglycemic conditions in ND7/23 DRG cells, and FK228 treatment was associated with a significant increase in pNrf2 expression (** *p* < 0.01, * *p* < 0.05; Figure 4d and Supplementary Figure S4d), suggesting a potential effect on oxidative stress-related pathways.

## 4. Discussion

Diabetic painful neuropathy (DPN) is a multifactorial complication characterized by metabolic dysfunction, oxidative stress, neuroinflammation, and progressive neuronal injury. Emerging evidence suggests that disruption of specialized neural barriers contributes to the pathogenesis of DPN by facilitating the entry of inflammatory mediators into neural tissues and exacerbating neuronal dysfunction [21,22]. Epigenetic mechanisms, particularly those regulated by histone deacetylases (HDACs), have been increasingly recognized as important modulators of neuronal survival, inflammation, and neuropathic pain [23]. Building upon our previous findings demonstrating the analgesic effects of the HDAC inhibitor FK228 in diabetic animals [9], the present study investigated whether FK228 treatment led to changes cell junction protein expression and stress-mediated signaling pathways in the spinal cord and dorsal root ganglia (DRG) under diabetic conditions.

The integrity of neural barriers depends on the coordinated expression of tight junction proteins, including claudins, occludin, and zona occludens (ZO) proteins [21,24]. Claudin-1 is a critical component of the blood–nerve barrier, while ZO-1 functions as a scaffolding protein that stabilizes interactions among tight junction complexes and the cytoskeleton [25]. Previous studies have shown that reductions in claudin and occludin expression following nerve injury are associated with increased barrier permeability, inflammatory cell infiltration, and the development of neuropathic pain behaviors [11,26]. Consistent with these observations, we observed that diabetic conditions are associated with altered expression of claudin-1, occludin, and ZO-1 in neural tissues. Importantly, FK228 treatment improved the expression of these junctional proteins, suggesting that HDAC inhibition may be associated with changes in tight junction protein expression in the diabetic nervous system. Although functional barrier permeability was not directly assessed in this study, the observed changes may extend the possibility that FK228 contributes to improving cell barrier protein expression under hyperglycemic conditions.

Neuronal degeneration and impaired regenerative capacity are hallmarks of diabetic neuropathy. GAP43 plays a central role in axonal growth, neuronal plasticity, and regeneration, and reduced GAP43 expression has been consistently reported in diabetic models [13,27]. Consistent with our previous work [9], hyperglycemic conditions significantly reduced GAP43 expression in ND7/23 DRG cells, indicating impaired regenerative potential. FK228 treatment increased GAP43 expression, suggesting activation of pathways involved in neuronal repair and axonal regeneration. Furthermore, we observed a significant reduction in NeuN expression in the DRG of diabetic animals, indicative of neuronal injury or atrophy. Increased NeuN expression following FK228 treatment further suggests preservation of neuronal subsistence in diabetic DRG after HDAC inhibition.

To further explore mechanisms underlying these effects, we examined EGFR signaling, which has been implicated in both neuropathic pain and regulation of tight junction formation [28]. Elevated EGFR activity has been reported in sensory neurons during neuropathic pain states, and pharmacological inhibition of EGFR reduces pain-related behaviors in experimental models [18]. EGFR signaling has also been linked to regulation of tight junction proteins and barrier permeability through downstream pathways including PI3K/Akt signaling [29,30]. In the present study, diabetic conditions were associated with increased EGFR expression in the spinal cord and DRG, whereas FK228 treatment significantly reduced EGFR levels. These findings are consistent with previous reports demonstrating that HDAC inhibition can suppress EGFR signaling [31]. Under normal physiological conditions, EGFR has been implicated in maintaining tight junction proteins such as occludin and claudin [18], which can be dysregulated due to hyperglycemic milieu. Even though the precise relationship between EGFR regulation and restoration of tight junction proteins requires further investigation, our data suggest that modulation of EGFR signaling may contribute to the positive effects of FK228 in diabetic neuropathy.

Oxidative stress is a major driver of neuronal dysfunction and progression of DPN. Nrf2 is a master regulator of cellular antioxidant defenses and has been shown to protect against oxidative injury and inflammation in diabetic tissues [15,32]. Impaired Nrf2 signaling has been associated with increased susceptibility to oxidative damage and neurodegeneration [33,34]. In agreement with previous reports, hyperglycemic conditions reduced Nrf2 activation in DRG cells. FK228 treatment increased Nrf2 phosphorylation, suggesting enhanced activation of endogenous antioxidant pathways. This observation increases the possibility that FK228 may reduce oxidative stress, at least in part, through antioxidant defense pathway and activation of Nrf2 may represent an important mechanism that would help protect neuropathy in diabetic condition.

Collectively, the findings of this study indicate that FK228 may influence several pathways relevant to diabetic neuropathy including changes in tight junction protein expression, neuronal survival, EGFR expression and Nrf2-mediated antioxidant pathways. These mechanisms may also associate with our previously observed findings with reduction of neuropathic pain following FK228 treatment. Nevertheless, several limitations are acknowledged. The study was performed in preclinical models and assessment of barrier permeability or inflammatory cytokine infiltration was not evaluated in relation to HDAC inhibition to junctional protein regulation. Future studies including functional barrier assays, translational investigations in human tissues may further define the therapeutic potential of FK228 for DPN.

## 5. Conclusions

In summary, the present study demonstrates that diabetic conditions are associated with disruption of tight junction protein expression, reduced neuronal regenerative capacity, increased EGFR signaling, and impaired Nrf2 activation in the peripheral nervous system. Treatment with the HDAC inhibitor FK228 may improve the expression of key junctional proteins, enhance the expression of the markers of neuronal survival and regeneration, reduce EGFR expression, and promote activation of antioxidant signaling pathways. While further studies are required to establish causality, evaluate functional barrier integrity, and determine clinical applicability, our findings suggest that HDAC inhibition warrants further investigation as a promising therapeutic strategy for DPN.

## Data Availability

All data generated or analyzed during the current study are included in this published article. Additional data are also available.

## Supplementary Materials

The following supporting information can be downloaded at: https://www.mdpi.com/article/10.3390/biology15151257/s1, Figure S1b. Complete Western blot image for HDAC2 with corresponding β-actin blot are shown in the left panel. Middle panel shows protein maker blot as well as the calculations for the Figure 1b. The right panel shows the Figure 1b as shown in the manuscript. Figure S1e. Complete Western blot image for Claudin-1 with corresponding β-actin blot are shown in the left panel. Middle panel shows protein maker blot as well as the calculations for the Figure 1e. The right panel shows the Figure 1e as shown in the manuscript. Figure S1f. Complete Western blot image for ZO-1 with corresponding β-actin blot are shown in the left panel. Middle panel shows protein maker blot as well as the calculations for the Figure 1f. The right panel shows the Figure 1f as shown in the manuscript. Figure S2b. Complete Western blot image for NeuN with corresponding β-actin blot are shown in the left panel. Middle panel shows protein maker blot as well as the calculations for the Figure 2b. The right panel shows the Figure 2b as shown in the manuscript. Figure S3b. Complete Western blot image for Claudin-1 with corresponding GAPDH blot are shown in the left panel. Middle panel shows protein maker blot as well as the calculations for the Figure 3b. The lower panel shows the Figure 3b as shown in the manuscript. Figure S3c. Complete Western blot image for occludin with corresponding GAPDH blot are shown in the left panel. Middle panel shows protein maker blot as well as the calculations for the Figure 3c. The right panel shows the Figure 3c as shown in the manuscript. Figure S3f. Complete Western blot image for ZO-1 with corresponding GAPDH blot are shown in the left panel. Middle panel shows protein maker blot as well as the calculations for the Figure 3f. The right panel shows the Figure 3f as shown in the manuscript. Figure S4a. Complete Western blot image for EGFR with corresponding GAPDH blot are shown in the left panel. Middle panel shows protein maker blot as well as the calculations for the Figure 4a. The lower panel shows the Figure 4a as shown in the manuscript. Figure S4b. Complete Western blot image for EGFR with corresponding GAPDH blot are shown in the left panel. Middle panel shows protein maker blot as well as the calculations for the Figure 4b. The right panel shows the Figure 4b as shown in the manuscript. Figure S4c. Complete Western blot image for EGFR with corresponding GAPDH blot are shown in the left panel. Middle panel shows protein maker blot for the Figure 4c. The right panel shows the calculations as well as the Figure 4c as shown in the manuscript. Figure S4d. Complete Western blot image for p-Nrf2 with corresponding β-actin blot are shown in the left panel. Middle panel shows protein maker blot as well as the calculations for the Figure 4d. The right panel shows the Figure 4d as shown in the manuscript.

## Abbreviations

The following abbreviations are used in this manuscript:

DRG	Dorsal root ganglia
DPN	Diabetic painful neuropathy
HDAC	Histone deacetylase
T2D	Type 2 diabetes
GAP43	Growth-associated protein 43
EGFR	Epidermal growth factor receptor
FK228	Romidepsin, HDAC inhibitor
Nrf2	Nuclear factor E2-related factor 2
