# Supplementary material for: FK228-Mediated Restoration of Tight Junction Proteins in Diabetic Neuropathy
Source: Biology (Basel). 2026 Jul 31;15(15):1257. doi: 10.3390/biology15151257 (PMC13464615; doi:10.3390/biology15151257)
Supplement: Supplementary file 1 [file biology-15-01257-s001.zip › Supplementary Figure Legends.pdf]

Fig. S1b. Complete Western blot image for HDAC2 with corresponding β-actin blot are shown in the left panel. Middle panel shows protein maker blot as well as the calculations for the Fig. 1b. The right panel shows the Figure 1b as shown in the manuscript.

Fig. S1e. Complete Western blot image for Claudin-1 with corresponding β-actin blot are shown in the left panel. Middle panel shows protein maker blot as well as the calculations for the Fig. 1e. The right panel shows the Figure 1e as shown in the manuscript.

Fig. S1f. Complete Western blot image for ZO-1 with corresponding β-actin blot are shown in the left panel. Middle panel shows protein maker blot as well as the calculations for the Fig. 1f. The right panel shows the Figure 1f as shown in the manuscript.

Fig. S2b. Complete Western blot image for NeuN with corresponding β-actin blot are shown in the left panel. Middle panel shows protein maker blot as well as the calculations for the Fig. 2b. The right panel shows the Figure 2b as shown in the manuscript.

Fig. S3b. Complete Western blot image for Claudin-1 with corresponding GAPDH blot are shown in the left panel. Middle panel shows protein maker blot as well as the calculations for the Fig. 3b. The lower panel shows the Figure 3b as shown in the manuscript.

Fig. S3c. Complete Western blot image for occludin with corresponding GAPDH blot are shown in the left panel. Middle panel shows protein maker blot as well as the calculations for the Fig. 3c. The right panel shows the Figure 3c as shown in the manuscript.

Fig. S3f. Complete Western blot image for ZO-1 with corresponding GAPDH blot are shown in the left panel. Middle panel shows protein maker blot as well as the calculations for the Fig. 3f. The right panel shows the Figure 3f as shown in the manuscript.

Fig. S4a. Complete Western blot image for EGFR with corresponding GAPDH blot are shown in the left panel. Middle panel shows protein maker blot as well as the calculations for the Fig. 4a. The lower panel shows the Figure 4a as shown in the manuscript.

Fig. S4b. Complete Western blot image for EGFR with corresponding GAPDH blot are shown in the left panel. Middle panel shows protein maker blot as well as the calculations for the Fig. 4b. The right panel shows the Figure 4b as shown in the manuscript.

Fig. S4c. Complete Western blot image for EGFR with corresponding GAPDH blot are shown in the left panel. Middle panel shows protein maker blot for the Fig. 4c. The right panel shows the calculations as well as the Figure 4c as shown in the manuscript.

Fig. S4d. Complete Western blot image for p-Nrf2 with corresponding β-actin blot are shown in the left panel. Middle panel shows protein maker blot as well as the calculations for the Fig. 4d. The right panel shows the Figure 4d as shown in the manuscript.
